# Detection and Management of Intraoperative Pneumothorax during Laparoscopic Cholecystectomy

**DOI:** 10.1155/2020/9273903

**Published:** 2020-04-07

**Authors:** Mohammed Heyba, Areej Rashad, Abdul-Aziz Al-Fadhli

**Affiliations:** ^1^Kuwait Board of Anesthesiology, Kuwait City, Kuwait; ^2^Department of Anesthesia and Intensive Care, Farwaniya Hospital, Sabah Al Nasser, Kuwait; ^3^Department of General Surgery, Farwaniya Hospital, Sabah Al Nasser, Kuwait

## Abstract

Intraoperative pneumothorax is a rare but potentially lethal complication during general anesthesia. History of lung disease, barotrauma, and laparoscopic surgery increase the risk of developing intraoperative pneumothorax. The diagnosis during surgery could be difficult because the signs are often nonspecific. We report a case of a middle-aged gentleman who developed right pneumothorax during an elective laparoscopic cholecystectomy. The patient had no risk factors for adverse events during the preoperative assessment (ASA1). The patient underwent general anesthesia and was put on mechanical ventilation. The first signs of abnormality immediately after surgical port insertion were tachycardia and low oxygen saturation in addition to sings of airway obstruction. The diagnosis of pneumothorax was made clinically by chest auscultation and later confirmed by intraoperative chest radiograph. Supportive treatment was started immediately through halting the surgery and manually ventilating the patient using 100% oxygen. Definitive treatment was then done by inserting an intercostal tube. After stabilizing the patient, the surgery was completed; then, the patient was extubated and shifted to the surgical ward. Postoperative computed tomography (CT) scan was done and showed only minimal liver laceration. The patient was discharged after removing the intercostal tube and was stable at the follow-up visit. Therefore, it is important to have a high index of suspicion to early detect and treat such complication. In addition, good communication with the surgeon and use of available diagnostic tools will aid in the proper management of such cases.

## 1. Introduction

Pneumothorax is a rare but a potentially fatal intraoperative complication. Early detection and diagnosis of pneumothorax during general anesthesia could be challenging, as changes in the hemodynamic parameters can be nonspecific [1]. Patient-related risk factors of intraoperative pneumothorax are similar to those of spontaneous pneumothorax and include the presence of emphysematous bullae or blebs that may rupture with positive pressure ventilation [1, 2]. Intraoperative risk factors for developing intraoperative pneumothorax include surgical manipulation of areas close to the parietal pleura, including intrathoracic surgeries, as well as central venous line placement [3, 4]. In addition, pneumoperitoneum is a reported contributor to pneumothorax. In fact, pneumothorax is an established complication of laparoscopic surgery, with a reported incidence of 0.01–0.4% [5–7]. Rarely, some cases of pneumothorax were associated with airway manipulation, though in these cases, there were other risk factors for pneumothorax [8, 9]. The recognition of pneumothorax can be difficult during general anesthesia, and some cases are only identified postoperatively [10].

Here we report a case of a healthy middle-aged gentleman who was planned for elective laparoscopic cholecystectomy under general anesthesia, who developed pneumothorax during the surgery. We describe the intraoperative management and the postoperative course of the patient.

## 2. Case

**Figure 1:**
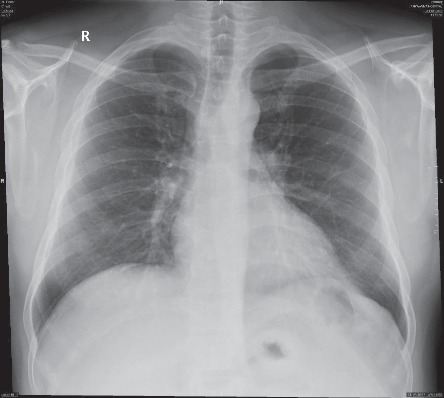
Preoperative chest radiograph for the patient showing normal lung fields with mild increase in bronchocovascular markings on the right lung.

Informed consent was obtained from the patient to publish this case report. A 42-yr-old gentleman was admitted as a case of cholelithiasis and planned for elective laparoscopic cholecystectomy. Preoperative assessment showed the patient to be previously healthy, with no past history of smoking, and the patient had normal physical examination. The routine laboratory investigations, including the full blood count, renal, and liver function tests, were within normal limits. Chest X-ray was normal except for mild increase in the basal bronchovascular markings on the right lung (Figure 1). Electrocardiogram (ECG) was done and was normal; abdominal ultrasound was done showing multiple gallbladder stones in an otherwise unremarkable study. The patient was planned for general anesthesia as an ASA-1.

**Figure 2:**
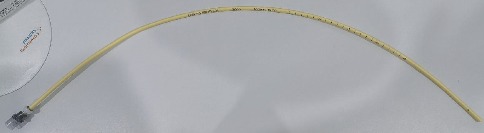
Airway exchange catheter (AEC) used to change the endotracheal tube (ETT).

Induction of general anesthesia was done using intravenous injection of propofol and fentanyl, and anesthesia was maintained with inhalation of sevoflurane. Muscle relaxation was achieved using intravenous pancuronium. The patient had a Cormac stage 2 by direct laryngoscopy. The patient was intubated using a size 8 endotracheal tube; however, inflation of the cuff was not achievable. It was presumed that there was a leak from the tube cuff, so the endotracheal tube was exchanged using an airway exchange catheter (AEC) (Figure 2), and the tube was fixed at the 22 cm mark. Proper tube placement was then confirmed by lung auscultation, which indicated equal air entry bilaterally, and all the vital signs were within normal. The patient was then connected to mechanical ventilation, using the pressure-control volume-guaranteed mode, with a tidal volume of 500 ml, respiratory rate of 12, and positive end-expiratory pressure (PEEP) of 8 mmHg. The airway pressure was maintained under 18 mmHg. The surgeon then proceeded with the placement of the ports and peritoneal insufflation.

**Figure 3:**
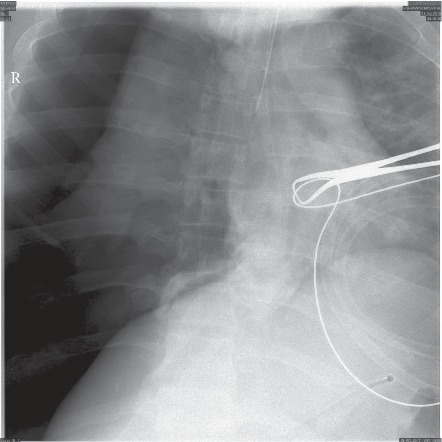
Intraoperative chest radiograph showing right-sided pneumothorax.

**Figure 4:**
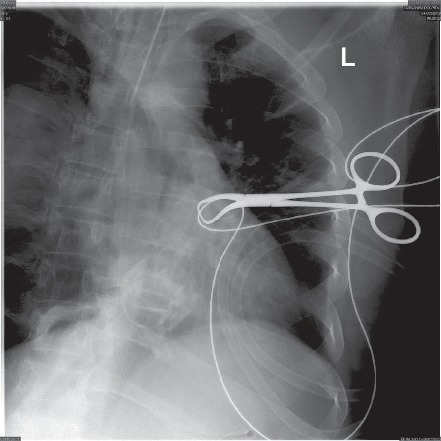
Intraoperative chest radiograph showing normal left lung field.

After achieving pneumoperitoneum and insertion of the laparoscopic camera and then the epigastric surgical port, the patient started to show signs of increased airway pressure. The airway pressure raised to over 30 mmHg and the capnogram started to show a delayed rise, indicating airway obstruction. The surgeon also indicated that he was not able to visualize the gallbladder properly as the diaphragm was displaced downwards. Within less than one minute, the patient heart rate started to increase reaching 120 Bpm; the oxygen saturation started to drop reaching 40%, but the blood pressure was still within normal. At that point, the surgeon was asked by the anesthetist to halt the surgery, the abdomen was deflated, and the anesthetist started the emergency management. The anesthetist confirmed the patency and placement of the endotracheal tube and then put the ventilator on manual mode with 100% oxygen high flow. Manual bag ventilation succeeded in restoring the oxygen saturation to over 90% in less than 30 seconds. Auscultation revealed decreased air entry over the right lung field. At that point, we suspected the presence of right-side pneumothorax and the surgical team was asked to prepare for intercostal tube insertion. The intraoperative X-ray machine (The C-arm) was requested immediately to the operating room and the chest image confirmed the presence of right-sided pneumothorax (Figure 3). Another image was taken to the left lung which showed the left lung to be normal. The surgeon then inserted an intercostal tube immediately on the right side, and another chest X-ray taken afterwards showed satisfactory lung expansion (Figure 4). The patient was hemodynamically stable, and the surgeon proceeded with laparoscopic cholecystectomy. The patient was extubated after the surgery with the intercostal tube in place and was maintaining satisfactory oxygen saturation on room air.

**Figure 5:**
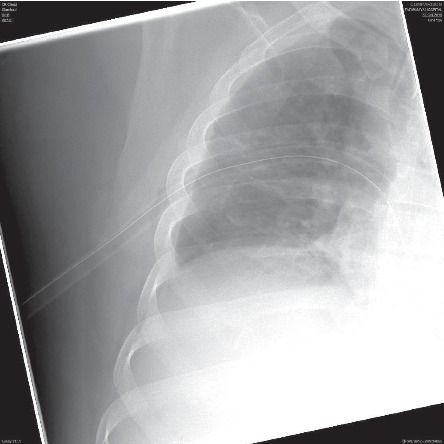
Intraoperative chest radiograph after insertion of intercostal tube showing normal lung expansion.

**Figure 6:**
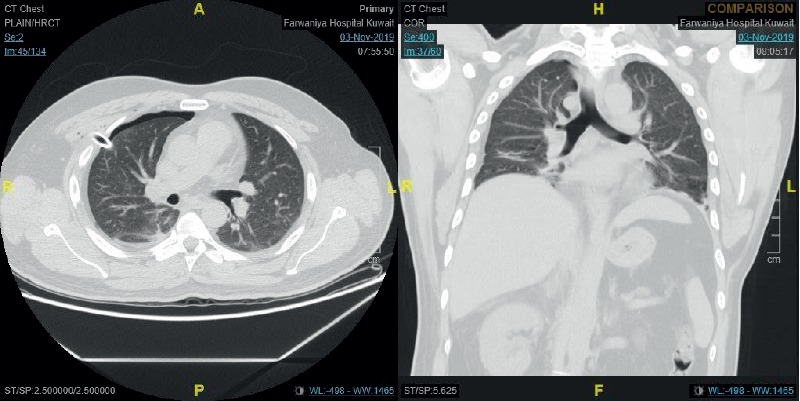
Postoperative computed tomography (CT) scan showing minimal right-side residual pneumothorax and the intercostal tube in place.

**Figure 7:**
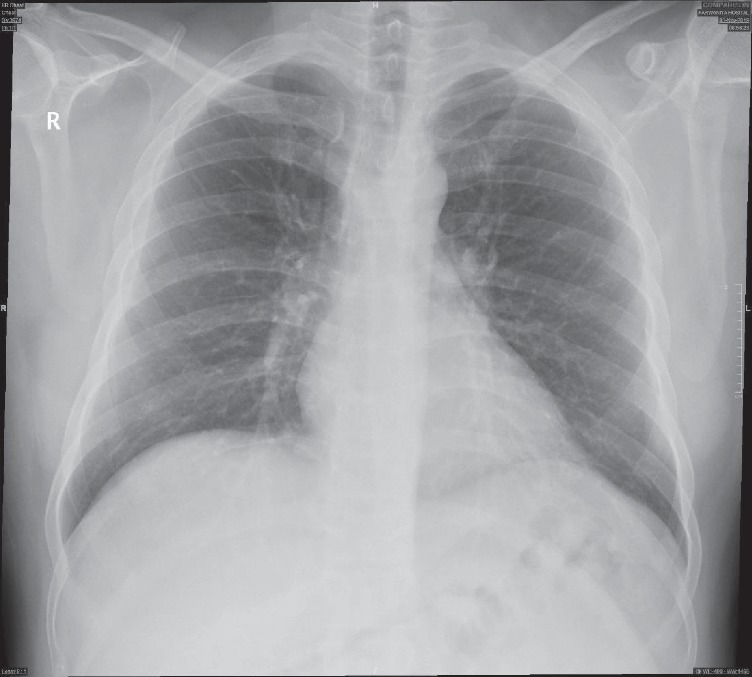
Follow-up chest radiograph showing no evidence of recurrence of pneumothorax.

The patient was discharged to the surgical ward, and follow-up chest X-ray was done on the following day, showing proper intercostal tube placement and proper lung expansion (Figure 5). Computed tomography (CT) scan was done on postoperative day 2 showing minimal pneumothorax, normal architecture of the trachea, and the bronchial tree, no evidence of bullae, no evidence of pneumomediastinum, and a right hepatic laceration (Figure 6). The intercostal tube was removed on postoperative day 3 (Figure 7), and the patient was discharged home on day 4 postoperatively. The patient was seen on a follow-up visit two weeks later and was hemodynamically stable without clinical or radiological evidence of recurrence of the pneumothorax.

## 3. Discussion

Pneumothorax during general anesthesia is a rare event, but can potentially be life threatening, especially if it develops into tension pneumothorax [11]. The major risk factor for developing pneumothorax during general anesthesia is positive pressure ventilation [12]. It is known that increasing the positive end-expiratory pressure “PEEP” increases the risk of barotrauma, especially in presence of respiratory distress syndrome (ARDS) [13]. The presence of emphysematous bullae or smaller blebs is a well-known situation in which pneumothorax develops with positive pressure ventilation due to bullae or blebs rupture [14]. The current recommendation is to avoid positive pressure during the ventilation of patients with known emphysematous lung disease, including bullae and pleural blebs [15]. Previous cases reported the development of pneumothorax in mechanically ventilated patients under general anesthesia, in which the presence of bullae was the main reason for the development of pneumothorax [15]. Single lung ventilation, either intentional or accidental, might lead to the development of pneumothorax with positive pressure ventilation, especially in the presence of small blebs [16]. In our patients, there was no reason to suspect he had an emphysematous lung bulla in the preoperative assessment chest radiograph. Additionally, no bullae were seen postoperatively by chest computed tomography (CT) scan. In our patient, we did use positive pressure ventilation; additionally, the endotracheal tube might have been located in the right main bronchus (Figure 3), which might have led to single lung ventilation and alveolar or bleb damage with mechanical ventilation at some point during the operation. The tube, however, appears to be retracted in the trachea in the radiograph taken afterwards (Figure 4); therefore, we cannot confirm or exclude that this is the cause of pneumothorax. Although no bullae were seen in the postoperative CT scan, this does not exclude the presence of small blebs or alveolar tears, which would not always be seen on CT scans [12, 14, 17].

Laparoscopic surgery has negative effects on ventilation because of carbon dioxide insufflation; these effects include decreased functional residual capacity, hypercapnia, and increase ventilatory pressure. Therefore, pneumothorax during laparoscopic surgery is especially potentially life threatening as it exacerbates these effects [18, 19]. Laparoscopic surgery itself is considered a risk factor for developing pneumothorax [20]. Factors that contribute to the development of pneumothorax during laparoscopy include leak of carbon dioxide from the peritoneal cavity into the pleural cavity either by diffusion or through a congenital defect in the diaphragm [18, 20]. Another risk factor related to laparoscopic surgery is diaphragmatic injury, due to surgical port insertion through the diaphragm, or through direct damage during manipulation of the liver during dissection of the gallbladder from its bed [19, 21, 22]. In our patient, the presence of right lobe liver laceration that was found in the postoperative CT scan might indicate surgical injury to the diaphragm; however, the presence of large diaphragmatic injury is unlikely because it would have been difficult to achieve pneumoperitoneum after intercostal tube insertion [23, 24]. In such case, the air from pneumoperitoneum would continue to leak through the diaphragmatic injury to the thoracic cavity and then through the intercostal tube to the underwater drainage system; this did not happen in our case, in which pneumoperitoneum was achieved successfully after intercostal tube insertion. On the other hand, a small diaphragmatic injury, caused by dissection instruments or Veress needle, for instance, which can heal spontaneously [25], could have happened in our patient. The presence of diaphragmatic injury could not be confirmed or excluded by the postoperative CT, as the gold standard for diagnosing and treating diaphragmatic injury is surgical exploration and repair, either open or laparoscopic [23, 25]. In our patient, the surgeons did not report observing any diaphragmatic injuries afterwards during laparoscopy.

Another rare factor that might increase the risk of developing pneumothorax is excessive airway manipulation during tracheal intubation [8, 26, 27]. Previous studies reported the development of pneumothorax with surgical instrumentation of the trachea during percutaneous tracheostomy. This could be explained by the damage to the tracheobronchial tree and leakage of air through the peritracheal tissue into the pleural space [28]. In these cases, the development of pneumothorax was often associated with pneumomediastinum or subcutaneous emphysema [28, 29]. Another scenario in which airway manipulation could lead to the development of pneumothorax is the use of airway exchange catheter (AEC) [8]. Airway exchange catheters were introduced to facilitate re-intubation in patients whom airway is deemed difficult for intubation [30]. The AEC is a hollow tube that can be passed through the lumen of the endotracheal tube (ETT), then the ETT can be removed, and another one introduced over the AEC with ease [30]. In some models, ventilation can be done through the AEC itself [31]. The use of AEC has been associated with some complications reported in the literature; among these complications is the development of pneumothorax [32]. It is theorized that pneumothorax can develop with the use of AEC due to injury to the tracheobronchial tree by the tip of the AEC or due to barotrauma with the use of jet ventilation [31]. In almost all cases of tracheobronchial tree injury caused by AEC use, there is an associated pneumomediastinum [26]. Bronchoscopy is the only investigation that can confirm the diagnosis of tracheobronchial rupture, but it is not always indicated [26]. Minor injuries to the tracheobronchial tree that can be managed by intercostal tube insertion and injuries that do not have a progressive course usually do not require bronchoscopic evaluation, and follow-up is the usual course of action [26, 29]. In some cases where broncheopleural air leak continues, subcutaneous emphysema or pneumomediastinum progresses, and lung expansion cannot be achieved after insertion of intercostal tube, bronchoscopy and surgical repair of the tracheobronchial injury is indicated [26, 33]. In our patient, we did not use jet ventilation through the AEC during tube exchange. Additionally, there was no evidence of injury to the tracheobronchial tree or evidence of pneumomediastinum in the postoperative CT; this, however, does not exclude the presence of minor injuries to the tracheobronchial tree, which might not be seen on CT in some cases [34, 35].

In many cases of pneumothorax during laparoscopy, the exact mechanism of development of pneumothorax might not be identified [20] as in our patient. Regardless of the cause of pneumothorax, early recognition and management is crucial for preventing unfavorable outcomes, as any pneumothorax can become tension pneumothorax with positive pressure ventilation used during anesthesia [1]. This requires having a high index of suspicion and alertness to any change in the vital signs of the patient. In most cases, the diagnosis of pneumothorax during anesthesia is that of exclusion, because many of the signs and changes in the vital signs of the patient are nonspecific [1]. Classically, diagnostic algorithms for crisis management are used to detect life-threatening complications during anesthesia. One of the most known such algorithms is the structured “core” algorithm (based on the mnemonic COVER ABCD–A SWIFT CHECK) [36]. This algorithm is a quick checklist that the anesthetist uses in a sequence to identify the cause of any deterioration in vital signs. This algorithm is claimed to diagnose and treat the problem in 60% of cases under general or regional anesthesia and provide a functional diagnosis in the remaining 40%. Case reviews showed that using this algorithm would have led to early recognition and better management of 12% of pneumothorax cases reported in the literature [1]. In our patient, the use of this algorithm, aided with the good communication with the surgeon [37] and the presence of portable X-ray machine in the operating room, led to early recognition and prompt management of the case. The surgeon noticed that the diaphragm was pushed downward after introducing the laparoscopic camera before any deterioration of the vital signs happened. After noting that there was decrease in the air entry to the right lung, the preparation to insert an intercostal tube was started and the portable X-ray machine (the *C*-arm) was called into the operating room while the patient was being stabilized. The diagnosis was then confirmed by the radiograph, although proper management was initiated immediately even before the diagnosis was confirmed.

The management of patient deterioration should start even before a specific diagnosis is confirmed. The initial management of any deterioration in vital signs should go simultaneously while diagnosing the main problem. The initial steps of management include immediate cessation of the surgery, deflation of the abdomen while the anesthetist is running the crisis algorithm. The patient is switched to manual ventilation with 100% oxygen once airway patency is confirmed. These were the steps taken in our case. The definitive management of pneumothorax, in the absence of tension pneumothorax, is the insertion of an intercostal tube. In our patient, once the vital signs were stabilized and the diagnosis of pneumothorax was confirmed, the surgeon was asked to insert an intercostal tube immediately. Afterwards, lung expansion was confirmed by taking another chest radiograph. After rechecking that all the vitals are stable and the patient was switched back to mechanical ventilation without difficulty, the surgeon proceed with the laparoscopic cholecystectomy. The decision of whether to proceed with the surgery should only be done after diagnosing and treating the problem and it should take into consideration the hemodynamic status of the patient and the urgency of the operation [38]. Although in our case the operation was an elective one, the patient was deemed stable and fit to undergo the surgery, which is what was decided.

## 4. Conclusion

In conclusion, pneumothorax is a rare but potentially lethal complication that can happen secondary to several reasons. Early recognition and management are crucial for preventing life-threatening complications. Using information aided by the surgeon from the surgical field of view and the availability of diagnostic tools, such as portable radiography machines, are valuable tools to facilitate early diagnosis and management of this problem.
